# Carvacrol Essential Oil: A Natural Antibiotic against Zoonotic Multidrug-Resistant *Staphylococcus* Species Isolated from Diseased Livestock and Humans

**DOI:** 10.3390/antibiotics10111328

**Published:** 2021-10-30

**Authors:** Ahmed H. Abed, Esraa F. Hegazy, Sherif A. Omar, Rehab M. Abd El-Baky, Ahmed A. El-Beih, Ahmed Al-Emam, Ahmed M. S. Menshawy, Eman Khalifa

**Affiliations:** 1 Bacteriology, Mycology and Immunology Department, Faculty of Veterinary Medicine, Beni-Suef University, Beni-Suef 62511, Egypt; esraahegazysayed@gmail.com; 2 Microbiology Department, Faculty of Veterinary Medicine, Cairo University, Cairo 12211, Egypt; sherif.marouf@cu.edu.eg; 3 Department of Microbiology and Immunology, Faculty of Pharmacy, Deraya University, Minia 11566, Egypt; rehab.mahmoud@mu.edu.eg; 4 Department of Microbiology and Immunology, Faculty of Pharmacy, Minia University, Minia 61519, Egypt; 5 Chemistry of Natural & Microbial Products Department, National Research Centre, Dokki, Giza 12622, Egypt; 6 Department of Pathology, College of Medicine, King Khalid University, Abha 61421, Saudi Arabia; amalemam@kku.edu.sa; 7 Department of Forensic Medicine and Clinical Toxicology, Faculty of Medicine, Mansoura University, Mansoura 35516, Egypt; 8 Department of Veterinary Medicine (Infectious Diseases), Faculty of Veterinary Medicine, Beni-Suef University, Beni-Suef 62511, Egypt; elmenshawy81@yahoo.com or; 9 Department of Microbiology, Faculty of Veterinary Medicine, Matrouh University, Matrouh 51511, Egypt; khalifa.eman@alexu.edu.eg

**Keywords:** *Staphylococci*, *S. aureus*, antimicrobial resistance, MRSA, VRSA, haemolysis, biofilm, resistance genes, virulence genes, carvacrol

## Abstract

*Staphylococcus* species cause diseases in animals and humans. The prevalence and antimicrobial profiles of *Staphylococcus* spp. in animals and human samples in the Minya Governorate, Egypt, were determined, and resistance- and virulence-associated genes were observed in multidrug-resistant (MDR) isolates. Moreover, the antibacterial effect of carvacrol essential oil (EO) on the MDR isolates was studied. A total of 216 samples were aseptically collected from subclinically mastitic cow’s milk (*n* = 100), sheep abscesses (*n* = 25) and humans (*n* = 91). Out of 216 samples, a total of 154 single *Staphylococcus* species (71.3%) were isolated. The most frequent bacterial isolates were *S. aureus* (43%), followed by *S. schleiferi* (25%), *S. intermedius* (12%), *S. xylosus* (12%), *S. haemolyticus* (4.5%), S. *epidermidis* (2%) and *S. aurecularis* (1%). Haemolytic activity and biofilm production were detected in 77 and 47% of isolates, respectively. Antimicrobial susceptibility testing showed a high degree of resistance to the most commonly used antimicrobials in human and veterinary practices. The *mec*A, *van*A, *van*C1 and *erm*C resistance genes were detected in 93, 42, 83 and 13% of isolates, respectively. Moreover, *hla*, *ica*A and *ica*D virulence genes were detected in 50, 75 and 78% of isolates, respectively. Carvacrol effectively inhibited the growth of all tested isolates at concentrations of 0.1, 0.05 and 0.04% while a concentration of 0.03% inhibited 75% of isolates. Interestingly, some phenotypic changes were observed upon treatment with a carvacrol oil concentration of 0.03%. All the treated MDR *Staphylococcus* isolates changed from multidrug resistant to either susceptible or intermediately susceptible to 2–3 antimicrobials more than parental bacterial isolates. Real-time PCR was applied for the detection of the differential expression of *mec*A and *van*C1 genes before and after treatment with carvacrol which revealed a mild reduction in both genes’ expression after treatment. *Staphylococcus* spp. Containing MDR genes are more likely to spread between humans and animals. From these results, carvacrol EO is a promising natural alternative to conventional antimicrobials for pathogens impacting human health and agriculture due to its potential antimicrobial effect on MDR pathogens; even in sub-lethal doses, carvacrol EO can affect their phenotypic properties and genes’ expression.

## 1. Introduction

The genus *Staphylococcus* comprises 81 species and subspecies with most members being ubiquitous versatile mammalian opportunistic pathogens that can colonize skin as well as diverse mucosal membranes. Several species are of significant medical or veterinary importance [1]. Their pathogenicity is mostly related to a combination of toxigenicity, invasiveness and antibiotic resistance [2].

Among the 250 potential causes of infectious bovine mastitis, the genus *Staphylococcus* is a principal etiological agent [3], in part because its high frequency and severe pathology and resulting disease [4]. In addition, *Staphylococcus* species—especially *S. aureus*—can lead to several infections in humans (e.g., soft tissue infection, impetigo, abscesses, necrotizing fasciitis, cellulitis [5], staphylococcal scalded skin syndrome, septic arthritis, endocarditis, pneumonia and meningitis [6]).

*Staphylococci* are among the most prominent of all nosocomial pathogens. Although *S. aureus* is clearly the primary pathogen, the coagulase-negative *Staphylococci* (CNS) colonizing both animal and human skin and mucous membranes are also capable of causing disease [7]. To date, more than 50 *Staphylococcus* species and subspecies have been implicated in bovine staphylococcal mastitis [8]. Due to the possible horizontal transmission of resistance genes [9] to other human pathogens or direct transmission to humans due to zoonotic virulent *Staphylococcus* species sharing between animal and human [10], the risks of *Staphylococci*-induced bovine subclinical mastitis (SCM) have been extended to public health. *S. aureus* is also considered a major foodborne pathogen with some strains producing food enterotoxins resulting in staphylococcal food poisoning [11]. *S. aureus* enterotoxins (Ses) are divided into five serological “classical types” (SEA, SEB, SEC, SED and SEE), however, new types of Ses and staphylococcal-like proteins have recently been described [2].

Instead of coagulase, *Staphylococci* can produce enzymes that allow them to invade host tissues and propagate the inflammatory process (e.g., lipase, fibrinolysin and urease). Additionally, they were discovered to be capable of producing proteolytic enzymes, exotoxins and haemolysins that aid in the uptake of iron such as staphylococcal protein A (SpA), the staphylococcal binder of immunoglobulin (Sbi), adenosine synthase A (AdsA), PVL (associated with staphylococcal skin and pulmonary infections), toxic shock syndrome toxin (TSST-1) and enterotoxins which lead to staphylococcal food poisoning. [7,12]. Apart from additional virulence factors, *Staphylococci* are protected from both the local and systemic host immunity [13].

The ability to form biofilm is an important virulence factor that allows *Staphylococci* to be organized into multilayered cell clusters embedded in a matrix of extracellular polysaccharide (slime) encoded by the *ica*A, *ica*B, *ica*C and *ica*D genes, [14]; this allows *Staphylococci* to persist unaffected by antimicrobials [15] and be resistant to host immunity [7].

Antimicrobial resistance in many pathogenic bacteria—including *Staphylococcus* spp.—can develop as a result of the overuse of antimicrobial compounds in veterinary medicine [16]. The mechanism of antimicrobial resistance include methicillin-resistant *S. aureus* (MRSA) encoded by *mec*A or *mec*C genes and responsible for resistance to β-lactams antibiotics [9,17] as well as vancomycin-resistant *S. aureus* (VRSA) encoded by *van*A or other van resistance genes [18,19,20]. Food animals and their environments are reservoirs of both resistant bacteria and resistance genes that can be transferred to humans by direct contact or via the food chain [21].

Essential oils (EOs) have been recognized for their potential antimicrobial activities due to their high hydrophobicity which enables them to cross the bacterial cell membranes leading to a loss of function, damage of proteins, lipids and organelles within the bacterial cell and consequently cell death [22]. Oregano oil and its major phenolic components, especially carvacrol oil, have a wide spectrum of antimicrobial activity [23]. The current study was designed to investigate the prevalence of different *Staphylococcus* spp. In livestock and human samples in Minya Governorate, Egypt, determining their AMR profile as well as detecting some resistance- and virulence-associated genes in multidrug-resistant (MDR) isolates. Additionally, the antibacterial effect of carvacrol EO on the MDR isolates of human and animal origin and the expression of resistance genes were examined.

## 2. Results

### 2.1. Prevalence of Staphylococcus Species

**Figure 1:**
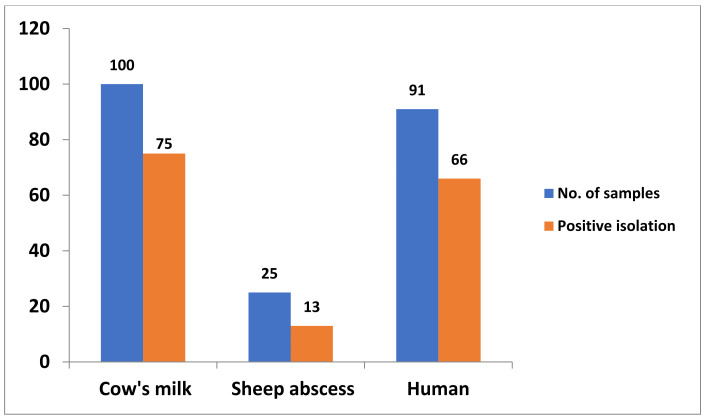
*Staphylococci* prevalence in the collected samples.

Out of 216 examined samples, 154 *Staphylococcus* spp. were isolated with a total prevalence of 71.3%. A total of 88 isolates were recovered from animals; 75 from 100 cow milk samples (75%) and 13 from 25 sheep abscesses (52%). Regarding the human samples (*n* = 91), 66 isolates (72%) were recovered. Wound and abscess samples (*n* = 30) showed the highest recovery rate, representing 22 isolates (73.3%), followed by dermal lesions (24/33; 72.7%) and tracheal samples (20/28; 71.4%) (Table 1 and Figure 1).

### 2.2. Identification and Prevalence of Different Staphylococcus spp. in Different Examined Samples

**Figure 2:**
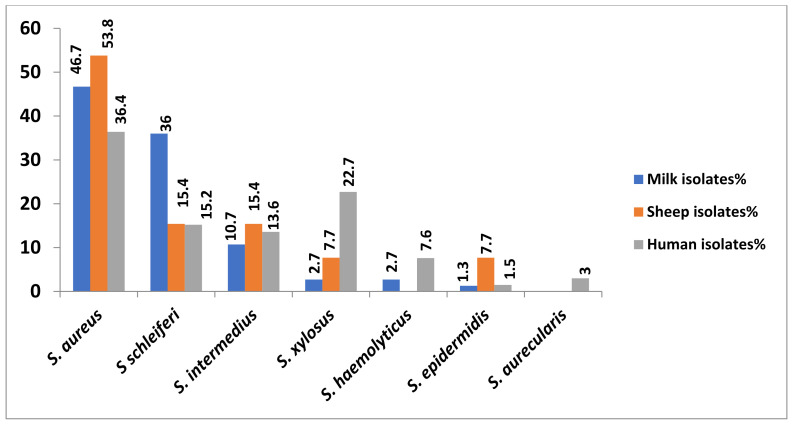
Prevalence of *Staphylococcus* spp. in different examined samples.

Out of 154 *Staphylococcus* species isolates, *S. aureus* was the most prevalent (66 isolates; 42.9%) followed by *S. schleiferi* (*n* = 39; 25.3%), *S. intermedius* (*n =* 19; 12.3%), *S. xylosus* (*n* = 18; 11.7%), *S. haemolyticus* (*n* = 7; 4.5%), S. *epidermidis* (*n* = 3; 1.9%) and finally *S. aurecularis* (*n* = 2; 1.3%) (Table 2 and Figure 2).

Regarding the animal isolates (*n* = 88), *S. aureus* was the most prevalent in the cow milk and sheep isolates with 35 (46.7%) and 7 isolates (53.8%), respectively, followed by *S. schleiferi* (27; 36% and 2; 15.4%, respectively), *S. intermedius* (8; 10.7% and 2; 15.4%, respectively) and *S. xylosus* (2; 2.7% and 1; 7.7%, respectively). Moreover, *S. haemolyticus* was identified in two milk isolates (2.7%) but was not found in any of the sheep isolates. Finally, one isolate of *S. epidermidis* was found in each sample (1.3 and 7.7%, respectively). Additionally, *S. aureus* was the most prevalent species in human isolates (*n =* 24; 36.4%) followed by *S. xylosus* (*n* = 15; 22.7%), *S. schleiferi* (*n =* 10; 15.2%), *S. intermedius* (*n =* 9; 13.6%), *S. haemolyticus* (*n* = 5; 7.6%), *S. aurecularis* (*n* = 2; 3%) and finally *S. epidermidis* (*n* = 1; 1.5%).

### 2.3. Haemolytic Activity and Biofilm Formation

**Figure 3:**
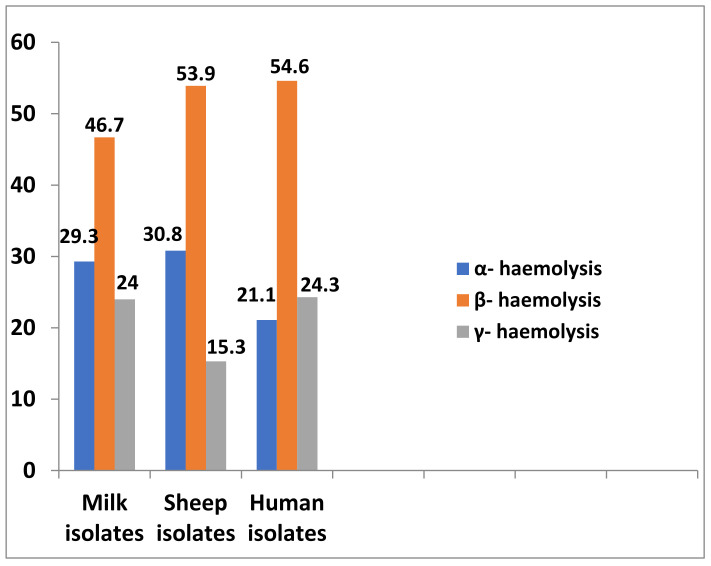
Types of haemolysis produced by *Staphylococcus* spp. isolated from different sources.

Out of 154 examined isolates, a total of 118 isolates (76.6%) were haemolytic. β-haemolysis was the most predominant, recorded in 78 isolates (50.6%), while α-haemolysis was recorded in 40 isolates (26%). On the other hand, 36 isolates (23.4%) were non-haemolytic (γ-haemolysis). In the same context, β-haemolysis was found in the milk, sheep and human isolates in representations of 46.7, 53.9 and 54.6%, respectively, while α-haemolysis represented 29.3, 30.8 and 21.1%, respectively. On the other hand, γ-haemolysis was recorded in 24, 15.3 and 24.3% of isolates, respectively (Table S1 and Figure 3).

**Figure 4:**
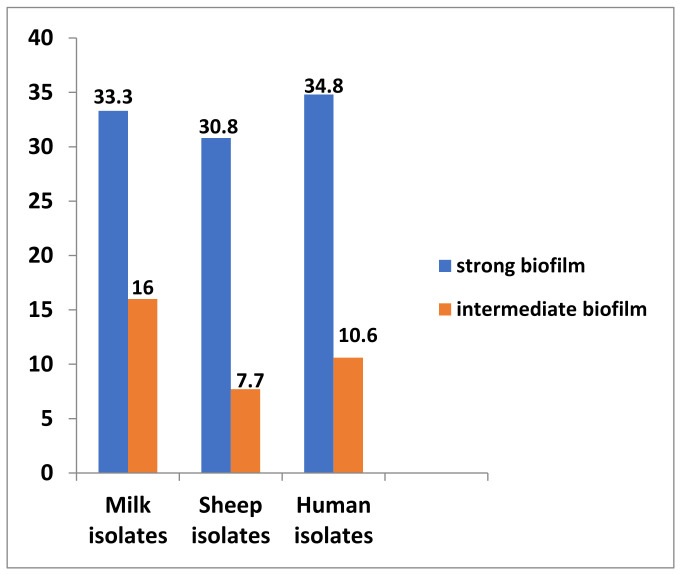
Biofilm formation produced by *Staphylococcus* spp. isolated from different sources.

For biofilm formation, a total of 72 isolates (46.8%) were phenotypically biofilm producer on Congo red agar (CRA) medium, among which 52 isolates (33.8%) were produced as strong and 20 isolates (13%) as intermediate. In contrast, 82 isolates (53.2%) not produced biofilm. Biofilm production represented 49.3, 38.5 and 45.5% in the milk, sheep and human isolates, respectively (Table S1 and Figure 4).

### 2.4. Antimicrobial Susceptibility Testing 

Regarding the milk *Staphylococcus* isolates, all were resistant to ampicillin, cefoxitin and amoxicillin–clavulanic acid. A high percentage of isolates were resistant to cefuroxime, clindamycin, chloramphenicol and vancomycin. On the other hand, all isolates were sensitive to imipenem while a high percentage of isolates were sensitive to ciprofloxacin and sulfamethoxazole–trimethoprim, while a moderate percentage of isolates were sensitive to azithromycin. All milk isolates (100%) were MDR and most were resistant to at least seven antimicrobials (Table S2).

All sheep isolates were resistant to ampicillin, cefoxitin, amoxicillin–clavulanic acid and clindamycin. Additionally, a high percentage of isolates were resistant to cefuroxime, vancomycin, kanamycin and chloramphenicol. On the other hand, all isolates were sensitive to imipenem while a high percentage of isolates were sensitive to ciprofloxacin, sulfamethoxazole–trimethoprim and azithromycin. All sheep isolates (100%) were MDR and most were resistant to at least six antimicrobials (Table S3).

For the human isolates, all were resistant to ampicillin, cefoxitin and amoxicillin–clavulanic acid. Additionally, a high percentage of isolates were resistant to clindamycin, kanamycin, cefuroxime, sulfamethoxazole–trimethoprim, azithromycin and ciprofloxacin. Meanwhile, a moderate percentage of isolates were resistant to chloramphenicol. On the other hand, most of the isolates were sensitive to imipenem while a moderate percentage of isolates were sensitive to vancomycin. All human isolates (100%) were MDR and most were resistant to at least eight antimicrobials (Table S4).

### 2.5. PCR Detection of Resistance- and Virulence-Associated Genes

**Figure 5:**
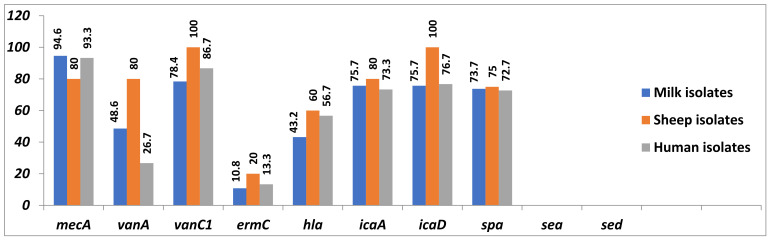
Prevalence and distribution of resistance- and virulence-associated genes in the examined Staphylococcus isolates.

The prevalence and distribution of resistance- and virulence-associated genes in MDR-, haemolytic- and biofilm-producing *Staphylococcus* isolates are shown in Table S5 and Figure 5. All isolates were confirmed as *Staphylococcus* spp. based on 16S rRNA gene analysis. The *mec*A, *van*A, *van*C1 and *erm*C resistance-associated genes were observed in a total of 67 (93.1%), 30 (41.7%), 60 (83.3%) and 9 isolates (12.5%), respectively. These genes represented in milk, sheep and human isolates, respectively, as follows: *mec*A (94.6, 80 and 93.3%), *van*A (48.6, 80 and 26.7%), *van*C1 (78.4, 100 and 86.7%) and *erm*C (10.8, 20 and 13.3%).

On the other hand, *hla*, *ica*A and *ica*D virulence-associated genes were detected in a total of 36 (50%), 54 (75%) and 56 isolates (77.8%), respectively. These genes represented, respectively, as follows: *hla* (43.2, 60 and 56.7%), *ica*A (75.7, 80 and 73.3%) and *ica*D (75.7, 100 and 76.7%).

In addition, the *spa* gene was found in 73.7, 75 and 72.7% of the tested *S. aureus* isolates, respectively, whilst no isolates harboured the *sea* and *sed* genes.

### 2.6. Carvacrol EO Antibacterial Effect and MIC for Multidrug-Resistant Staphylococcus Isolates

**Figure 6:**
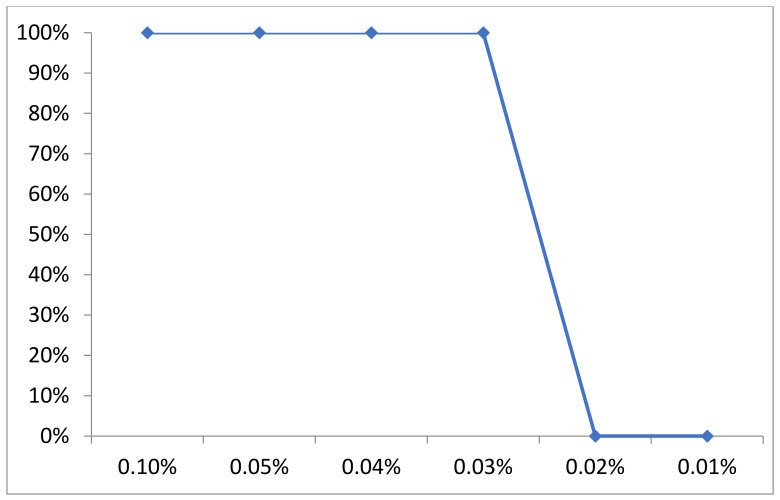
Carvacrol oil antibacterial effect on MDR *Staphylococcus* isolates.

Carvacrol EO inhibited the growth of all tested isolates (100%) at concentrations of 0.1, 0.05 and 0.04% (0.04% was considered the MIC). At a concentration of 0.03%, a total of 54 isolates (75%) were inhibited while 18 isolates (25%) were unaffected and distributed as nine *S. aureus*, four *S. schleiferi*, two *S. intermedius* and three *S. xylosus*. In contrast, the concentrations of 0.02 and 0.01% had no antibacterial effect with little retarded growth (Table 3 and Figure 6).

### 2.7. Carvacrol EO Antimicrobial Susceptibility with MDR Staphylococcus Isolates

All tested MDR *Staphylococcus* isolates (*n* = 18) were resistant to seven or eight different antimicrobial agents. The results of the re-evaluation of antimicrobial susceptibility after treatment with 0.03% carvacrol indicated that 11 isolates (61.1%) changed from being resistant to being susceptible or intermediately susceptible to three antimicrobial agents, while the seven other isolates (38.9%) changed to being susceptible or intermediately susceptible to two antimicrobials only.

### 2.8. MDR Staphylococcus Isolates’ Real-Time PCR Analysis before and after Treatment with Carvacrol Oil

The quantitative RT-PCR for unaffected MDR *Staphylococcus* isolates with the last concentration of carvacrol oil lower than the MIC (0.03%) before and after treatment showed a mild reduction in both the *mec*A and *van*C1 resistance genes’ expression after treatment and their fold changes ranged between 0.2–0.3-fold and 0.2–0.4-fold, respectively.

## 3. Discussion

This study investigated the prevalence of *Staphylococcus* spp. in animal (SCM cow milk and sheep abscesses) and human samples to determine AMR profiles—especially MRSA and VRSA—leading to the detection of some resistance- and virulence-associated genes in MDR isolates and studied the antibacterial effect of carvacrol EO on MDR isolates and resistance gene expression. In a similar study, different *Staphylococci* isolates from cows and humans were isolates with recovery rates of *Staphylococci* from mastitic cow milk and human samples of 40 and 30%, respectively [24]. In another study, the recovery rates for *S. aureus* were 21, 10 and 24% from the wounds and abscesses of cows, sheep and humans, respectively, and all isolates were MDR to 6–8 antimicrobials [25]. The similarity of *Staphylococcus* isolates recovered from humans and animals supported the theory that some animal Staphylococcal lineages are derived from human strains following genetic adaptation with a change in host specificity [26].

The bacteriological identification results of different *Staphylococcus* species isolated from different sources are similar to those Roberts et al. [27] who identified 19 different *Staphylococcus* spp. isolates from animal and human samples, among which *S. haemolyticus S. xylosus, S. saprophyticus*, *S. sciuri*, *S. succinus*, *S. chromogenes* and *S. aureus* were the most prevalent. Animal infections are harmful to health and can serve as a reservoir for Staphylococcal transmission to humans. Host-switching events between animals and humans and amongst animals are frequent and have been accentuated with the domestication and/or commercialization of specific animal species [1].

*Staphylococci* can produce many enzymes facilitating host tissues’ invasion and the spreading of the inflammatory process as well as the proteolytic enzymes and haemolysins which facilitate the uptake iron [28]. *Staphylococci* produce several haemolysins; alpha (α), beta (β), gamma (γ) and delta (δ), which are cytolytic exotoxins invading the host cell and degrading the cell membrane of erythrocytes, thus facilitating *Staphylococci* access to iron—specifically iron within haemoglobin [29]. In this study, a majority of isolates were haemolytic. Moraveji et al. [29] recorded haemolytic activity in 60 and 90% of bovine and human *Staphylococcus* isolates, respectively. Slime production and the ability of surfaces’ adherence to facilitate the biofilm formation is an important factor responsible for staphylococcal pathogenicity and intramammary survival [28]. Additionally, biofilms decrease antimicrobial susceptibility and impede antimicrobial therapy [30]. For the detection of biofilm in *Staphylococci*, CRA is adequate for routine use when run in parallel with PCR [31,32]. In this study, the isolates that were biofilm producers were consistent with the results found by El-Seedy et al. [28]. Moreover, Bochniarz et al. [12] observed a slime-producing ability in 54% of the recovered *Staphylococci* while higher values were recorded. [31,32,33].

The haemolytic activity and biofilm production of *Staphylococcus* isolates were genotypically assessed using PCR for the detection of *hla*, *ica*A and *ica*D genes. The *hla* gene was detected in half the tested isolates, which is lower than those recorded in previous studies for the *hla* gene in all *Staphylococcus* isolates (100%) [29,34]. These findings support the finding that the existence of the *hla* gene in *Staphylococci* is necessary to establish infection in animals and humans [29]. The synthesis of an extracellular slime component is generated by the genes of the *ica*RADBC locus, an operon of four biosynthetic genes, which is the primary step in biofilm development (*ica*ADBC) [31]. These genes are virulence markers for *Staphylococcus* spp. and their presence indicates the pathogenic potential of the strain [16]. In this study, *ica*A and *ica*D genes were detected in different *Staphylococcus* isolates. Osman et al. [31] observed *ica*A and *ica*D genes in 14 and 77% of *Staphylococcus* isolates, respectively, while Abed et al. [16] recorded the *ica*D gene in 13% of isolates. These findings suggest that biofilm formation requires a complex network of factors and that *ica*A and *ica*D genes are reliable gene markers for biofilm formation [16].

Antimicrobial treatment still represents the main control measures for human and animal infections. At the same time, the widespread use of antimicrobials is cited as the primary source of AMR [16]. Emerging antimicrobial resistance has been a global problem in recent decades, drawing increased attention to antimicrobial usage in animal agriculture and its possible impact on public health [21]. However, the role of agricultural antibiotic usage in the development and spread of human disease resistance is still being debated [35]. In the present study, the antimicrobial susceptibility profiles of *Staphylococcus* isolates from different sources revealed complete resistance to β-lactams, high resistances to clindamycin, cefuroxime, kanamycin and chloramphenicol and complete sensitivity to imipenem only. Phenotypic susceptibility to cefoxitin discs was employed for the estimation of methicillin-resistant *Staphylococci* (MRS) and a high incidence of MRS is characteristic in notorious *Staphylococci* leading to limited therapeutic options and successful antimicrobial therapy [16]. Moreover, most human isolates were resistant to sulfamethoxazole–trimethoprim, azithromycin and ciprofloxacin; whilst on the contrary, animal isolates were mostly sensitive to these. In contrast, most human isolates were vancomycin-sensitive while the animal isolates were vancomycin resistant. All animal and human isolates were MDR to 6–8 antimicrobials. Radwan et al. [25] reported relatively similar results but differed with regard to vancomycin resistance, which appeared in their animal isolates but not in those of human origin, and they also recorded a high rate of MDR *Staphylococcus* isolates of both animal and human origin while Schmidt et al. [36] recorded a lower rate of MDR *Staphylococcus* isolates of animal and human origin. El-Seedy et al. [28] and Abed et al. [17] also reported the same pattern in SCM *Staphylococcus* isolates. Different resistance against specific antimicrobials in *Staphylococcus* isolates of both animal and human origin may be attributed to the overuse of these antimicrobials in veterinary and human medicine. As animals are closely related to the environmental microbiome and resistome, staphylococcal strains of animal origin represent a source of resistance determinants [29]. The antimicrobial susceptibility profiles of *Staphylococcus* isolates from cattle and humans were found to be comparable, suggesting that animals and humans exchange *Staphylococcus* strains and that bovine MDR *Staphylococci* might be a zoonotic pathogen. Although it is difficult to confirm the direction of interspecies transmission, it has been speculated that *Staphylococci* are more likely to pass from people to dairy cattle than vice versa [9].

Where AMR is imparted by the presence of resistance genes, these can be connected to genetic elements and the usage of a particular antibiotic can select for resistance not just to that antimicrobial but also to others [16]. Methicillin resistance is conferred by a *mec*A gene found on the *Staphylococcal* chromosomal cassette (SCC) which encodes alternative penicillin binding proteins—PBP2a or PBP2ALGA—and enzymes that crosslink the peptidoglycans of the bacterial cell wall, reducing antibiotic binding to β-lactams [17,20]. Therefore, the acquisition of *mec*A promotes staphylococcal resistance to methicillin and other β-lactams [16]. Methicillin-resistant *Staphylococcu*s strains are of great public health significance as they mostly carry other resistance genes on a chromosome harbouring the *mec*A gene [36]. In this study, the *mec*A gene was found in a majority of isolates. The discovery that MRSA commonly colonizes animals, particularly livestock, has raised concerns about an enlarged reservoir of MRSA. While MRSA strains found in companion animals are typically identical to human nosocomial MRSA, MRSA strains found in food animals appear to be animal-adapted clones [26]. The high level of animal-emergent MRSAs, as well as the recent discovery of a divergent *mec*A MRSA that infects both livestock and humans, underscore the potential for farm animals to serve as a reservoir of infection for both other farm animals and the human population [37]. Vancomycin is one of the first-line drugs for the treatment of MRSA infections [20]. However, vancomycin-resistant isolates have emerged in recent years and have become a serious public health concern [19]. VRSA is mediated by a *van* gene cluster that is transferred from vancomycin-resistant enterococcus [20]. Although 11 *van* gene clusters confer vancomycin-resistance (*van*A, B, D, F, I, M, C, E, G, L and N phenotypes), only the *van*A gene cluster is responsible for the isolated VRSA strains [38]. In this study, the *van*C1 gene appeared more than *van*A. The characterization of these resistance genes reveals a strong association between the phenotypes and genotypes of AMR and their existence in animals is potentially hazardous to human health. This can occur through the lateral transfer of these genes between different *Staphylococcus* species and/or through direct infection with the resistant pathogens [38].

In *Staphylococci*, erythromycin resistance is frequently linked with resistance to other macrolides [39]. Three erythromycin ribosomal methylase genes (*erm*A, *erm*B and *erm*C) encoding methyltransferases that modify the ribosomal target site to confer resistance to macrolides, lincosamides and type B streptogramins (MLSB phenotype) have been discovered in *Staphylococci* [40]. In this study, *erm*C gene was found in only 12% of the isolates. Similar results were previously recorded [41,42] while Faezi et al. [40] reported that *erm*C and *erm*A were the two most dominant genes which occurred in 94–98% of erythromycin-resistant staphylococcal strains. The inconsistency of the phenotype–genotype association of AMR may be attributed to the presence of many other different genetic factors that stimulate the expression of resistance phenotypes and/or the possibility of other mechanism(s) such as the overexpression of efflux pumps, mutations or modifications in the target sites [16].

*SpA* plays a principal role in the pathogenesis of human and animal staphylococcal infections and binds the FC fragment of IgG impairing antibody functions [26]. The *spa* gene was mostly found in tested milk, sheep and human isolates, while *sea* and *sed* genes were not observed in any isolate. In some isolates, it was not possible to amplify the XR region of protein A encoded by the *spa* gene. This may be attributed to a complete absence of the *spa* gene or to deletions/insertions in the region encoding the IgG binding domain of protein A. This region is upstream of the XR region where it hybridizes the forward primer; thus, preventing amplification [43]. Baum et al. [44] explained the lack of amplification in some *S. aureus* isolates due to a deletion mutation (Deletion E at 174 bp) in the primer binding region of the *spa*-gene for the original forward primer. Votintseva et al. [45] also detected a deletion mutation; deletion G (63 bp) which always paired with insertion B (63 bp). Moreover, the absence of the amplification of the Xr region of the *spa*-gene in some *S. aureus* strains can be attributed to a weakness of the current *spa* primers due to rearrangements in the IgG-binding region of the gene and such rearrangements represented by deletion A (357 bp) and deletion D/insertion A (174 bp/10 bp) that do not affect the position of the standard forward primer [45]. In addition, Peacock et al. [46] used primers different to those used in the present study and reported the presence of different gene codes for virulence in natural populations of *S. aureus* and clarified that the effects of those genes were separately increasing the chances of disease and reported that it might be inaccurate to regard virulence in relation to the presence or absence of a given bacterial factor. Koreen et al. [47] suggested that repeat composition and organization allow SpA typing to correlate with the DNA microarray data. Recently, Brignoli et al. [48] suggested that the SpA-negative phenotype has occurred in geographically distinct strains through different molecular mechanisms including mutation, leading to likely translation alterations and transcriptional deregulation. Furthermore, there is evidence that SpA strains are highly susceptible to phagocytic uptake mediated by anti-capsule antibodies. These data suggest that *S. aureus* may alter its virulence factor expression pattern as an adaptation to the host or environment. Isolates with an identical sequence and *spa*-types are found in bovine and human isolates, indicating transmission between the two host populations [37].

Although antibiotics have been effective in the treatment of infectious diseases, resistance to these drugs has led to deleterious effects. The emergence and spreading of MDR microorganisms necessitate the discovery of new classes of antibacterial compounds that inhibit these resistance mechanisms. Natural drugs could represent an alternative approach [49]. Recently, medicinal herbs have been shown to be potential agents in the prevention and protection against infectious diseases and are safe in terms of human and animal health. Carvacrol is a significant component of essential oils and has recently attracted much attention as a result of its biological properties including a wide spectrum of antimicrobial activity [50]. The MIC of carvacrol oil for the tested *Staphylococcus* isolates was 0.04%, as observed for a variety of microorganisms [50,51,52,53,54]. In addition, carvacrol was suggested as the bioactive compounds against *S. aureus* and other Gram-positive and Gram-negative bacteria [51]. Moreover, de Souza et al. [53] evaluated the antimicrobial potential of carvacrol in vitro and in vivo in Swiss mice against MDR *K. pneumoniae* strains and indicated that the use of carvacrol as a therapeutic agent can exert significant in vitro and in vivo antimicrobial effects against carbapenemase-producing *K. pneumoniae* (KPC), increasing animal survival and significantly decreasing bacterial loads; such preliminary results in mice were hopeful and indicated that carvacrol has potential as an antimicrobial agent. Carvacrol can inhibit bacterial growth as a result of the disruption of the bacterial membrane integrity increasing its fluidity and permeability, resulting in inorganic ions and ATP leakage, pH homeostasis and cell death [50,51]. It also possesses antifungal and antibiofilm properties, thus it can be used as an antimicrobial alternative against MDR pathogenic bacteria [50]. The MIC of carvacrol against different bacteria was detected as 400µg/mL [49] while it was 125µg/mL against *S. pyogenes* [54].

Interestingly, some phenotypic changes of the AMS profile of unaffected MDR *Staphylococcus* isolates were observed upon treatment with the concentration lower than the MIC of carvacrol oil (0.03%). All the treated MDR *Staphylococcus* isolates were modified from resistant to either susceptible or intermediately susceptible to 2–3 antimicrobials more than the parental bacterial isolates. Real-time quantitative PCR showed the differential expression of *mec*A and *van*C1 resistance genes before and after treatment with carvacrol oil. No significant effect on either the detection or the expression of selected genes was observed where a mild reduction in both genes’ expression after treatment was seen. This study indicated that carvacrol and other EOs can serve as alternatives to antibiotics in the fight against pathogens that affect human health and agriculture. More research is needed to determine the impact of genetics and environmental variables on thymol and carvacrol concentration in the field.

## 4. Material and Methods

### 4.1. Samples

Samples (*n* = 216) were collected from animals (*n* = 125) and humans (*n* = 91) from January to November 2019 situated in the same locale: Minya Governorate, Egypt.

#### 4.1.1. Animal Samples

One-hundred fresh quart milk samples (QMSs) were aseptically collected from subclinically mastitic cows in two dairy farms with a history of SCM. Cows were mainly between their 3rd and 5th seasons of lactation after 2nd–6th months of calving. All of the collected QMS reacted positively when tested with the California mastitis test (CMT) according to Schalm et al. [55]

Moreover, 25 pus swabs were aseptically collected from sheep abscesses in two dairy farms.

#### 4.1.2. Human Samples

Ninety-one swabs were collected from human lesions including 30 abscesses and surgical wounds (15 abscesses and 15 surgical wounds; including 6 from diabetic foot, 4 appendicitis and 5 caesarean), 33 dermal lesions (12 acne, 10 impetigo, 5 pustule and 6 folliculitis) as well as 28 tracheal swabs from patients admitted to El-Minia University Hospital suffering from chronic obstructive pulmonary disease (COPD), pneumonia and other respiratory diseases (one sample from each patient).

All samples were immediately transferred to the laboratory in an icebox for bacteriological examination. 

### 4.2. Bacteriological Examination of the Collected Samples

Milk samples were centrifuged (3000 rpm, 15 min) and a loop was taken from the precipitate; the sample was inoculated on tryptone soy broth (Oxoid, Ltd., Basingstoke, Hampshire, UK) and incubated at 37 °C for 18–24 h. All swab samples were also inoculated onto tryptone soy broth and incubated. A loop aliquot from each broth culture was cultivated in 10% sheep blood agar, tryptone soya agar (TSA), mannitol salt agar and Baird–Parker agar (Oxoid, Ltd., Basingstoke, Hampshire, UK) and then incubated at 37 °C for 24–48 h. *Staphylococcus* species were suspected based on phenotypic characters of the colonies. Bacterial smears were prepared from the suspected colonies and stained with Gram’s stain [56].

### 4.3. Identification of Staphylococcus Isolates

*Staphylococcus* isolates were presumptively identified based on their Gram’s stain morphology and colonial features. Additional laboratory tests were used to confirm identification according to Quinn et al. [56], Waller et al. [57] and NMC [58]. Catalase, coagulase, oxidase, citrate utilization, Voges Proskauer, urease and mannitol fermentation assays as well as haemolysis on 10% sheep blood agar and pigment production were also employed. CNS isolates were also identified using Analytical Profile Index kits; API-Staph (BioMérieux, Marcy-l’Étoile, France). Following the manufacturer’s instructions, API strips were exclusively used to identify pure cultures. The Department of Bacteriology, Mycology, and Immunology of Faculty of Veterinary Medicine at Beni-Suef University, Egypt, provided the different positive control strains used for the API kits.

### 4.4. Antimicrobial Susceptibility Profiles of the Staphylococcal Isolates

Antimicrobial susceptibility (AMS) was based on a disc diffusion test with 12 distinct antimicrobial discs (Oxoid, Ltd., Basingstoke, Hampshire, UK) from various classes: ampicillin (10 mg), amoxicillin–clavulanic acid (30 mg), cefoxitin (30 mg), cefuroxime (30 mg), vancomycin (30 mg), imipenem (10 mg), ciprofloxacin (5 mg), azithromycin (15 mg), clindamycin (2 mg), kanamycin (30 mg), florfenicol (30 mg), florfenicol (30 g) and sulfamethoxazole–trimethoprim (25 µg). A phenotypic antimicrobial susceptibility test was performed on Muller–Hinton agar (Oxoid, Ltd., Basingstoke, Hampshire, UK) also using a disc diffusion method, with interpretations based on the CLSI guidelines [59]. AMS used breakpoints from the CLSI to create induced inhibition zones [59]. According to Chandran et al. [60], MDR was defined as resistance to three or more antimicrobials from separate antibiotic groups.

### 4.5. Phenotypic Detection of Slime Production (Biofilm Formation) on Congo Red Agar Medium

Biofilm production was phenotypically assessed by the Congo red agar (CRA) method as described by El-Seedy et al. [28]. All isolates were streaked onto the CRA medium, incubated at 37 °C for 24 h, and then kept at room temperature for 48 h. Colony colour was determined using a four-color reference scale varying from red to black. A positive result was indicated by black colonies while pink or purple colonies were considered negative. An indeterminate result was indicated by almost-black colonies.

### 4.6. Polymerase Chain Reaction

PCR-tested isolates were selected according to the results of antimicrobial susceptibility testing, haemolytic activities and biofilm production as they were MDR, haemolytic and biofilm producing isolates from different species of different sources distributed as 37 milk isolates (19 *S. aureus*, 11 *S. schleiferi*, 4 *S. intermedius*, 2 *S. xylosus* and 1 *S. haemolyticus*), 5 sheep isolates (4 *S. aureus* and 1 *S. schleiferis*) as well as 30 human isolates (11 *S. aureus*, 4 *S. schleiferi*, 4 *S. intermedius*, 7 *S. xylosus*, 3 *S. haemolyticus* and 1 *S. epidermidis*). The selected isolates were confirmed by the detection of staphylococcal 16S rRNA gene performed according to Mason et al. [61] and then screened for the detection of 4 resistance-associated genes (*mec*A, *van*A, *van*C1 and *erm*C) and 3 virulence-associated genes (*hla*, *ica*A, and *ica*D). In addition, the tested *S. aureus* isolates were investigated for 3 other virulence genes (*spa*, *sea* and *sed*). DNA extraction from samples proceeded according to the instructions for the QIAamp DNA micro kit (Cat. No. 51304). (Qiagen, Germany, GmbH). Table S6 [61,62,63,64,65,66,67,68,69,70] showed the primer sequences (Metabion, Germany), size of generated products, temperature and PCR time conditions. The PCR positive controls were provided by the Department of Bacteriology, Mycology, and Immunology of Faculty of Veterinary Medicine at Beni-Suef University, Egypt, and were completely identified strains known to carry the tested genes.

### 4.7. Agar Dilution Method for Detection of the Antibacterial Effect Carvacrol Essential Oils on Staphylococcus Isolates 

The antibacterial effect of different concentrations of carvacrol EO against 72 MDR *Staphylococcus* isolates was assessed by agar dilution method according to Radwan et al. [71]. Carvacrol (Sigma Aldrich, Germany) was dissolved in Dimethyl Sulpho Oxide (DMSO) at a ratio 1:9, filtered through a 0.45 µm cellulose filter membrane and then different concentrations (0.1, 0.05, 0.04, 0.03, 0.02 and 0.01%; volume/volume) were separately prepared by the addition of the oil to sterilize the cooled TSA (55 °C), before pouring 10 mL of oil–agar medium in sterile Petri dishes and left to solidify. The tested isolates were grown on TSA at 37 °C for 24 h and then the standard inoculums visually equivalent to the 0.5 McFarland standards (1.5 × 10^8^ CFU/mL) were prepared in physiological saline. Equal amounts of bacterial suspensions were inoculated and speared onto the entire surface of oil agar plates and incubated at 37 °C for 24–48 h. The incubated plates were examined for the growth or inhibition of the bacterial growth detecting the minimum inhibitory concentration (MIC) of carvacrol EO.

### 4.8. Phenotypic Effect of Carvacrol EO on Antimicrobial Susceptibility Profile of MDR Staphylococcus Isolates

Antimicrobial susceptibility testing was reapplied on those 18 MDR *Staphylococcus* isolates unaffected by carvacrol treatment at a concentration of 0.03%; the last concentration lower than MIC (as previously described in Section 4.7) was detected using the antimicrobial discs against which resistance was observed before and after treatment

### 4.9. SYBR Green RT-PCR on MDR Staphylococcus Isolates before and after Treatment with Carvacrol Oil

Quantitative SYBR Green RT-PCR was applied to a total of 18 MDR *Staphylococcus* isolates for the detection of *mec*A and *van*C1 resistance genes’ expression pre-exposure (control) and post-exposure to carvacrol EO; the 16S rRNA gene was used as a *housekeeping gene* for gene expression. The tested isolates were MDR isolates having both *mec*A and *van*C1 genes which were not affected by the minimum inhibitory concentration of carvacrol and distributed as 10 milk isolates (5 *S. aureus*, 3 *S. schleiferi*, 1 *S. intermedius* and 1 *S. xylosus*) 1 sheep isolate (*S. aureus*) as well as 7 human isolates (3 *S. aureus*, 1 *S. schleiferi*, 1 *S. intermedius* and 2 *S. xylosus*). RNA was extracted by RNeasy Mini Kit (Qiagen, Germany, GmbH Cat. No.74104) according to the manufacturer’s instructions. The SYBR green RT-PCR reaction volume was 25 μL—consisting of 12.5 μL of Quantitect SYBR green PCR master mix (Takara, Japan), 0.25 μL of RevertAid Reverse Transcriptase (Thermo Fisher), 0.5 μL of each forward and reverse primers, 3 μL of template DNA and 8.25 μL of PCR grade water. The cycling conditions of the different primers during RT-PCR are illustrated in Supplementary Table S7. The amplification curves and Ct values were determined by the Stratagene MX3005P software (Agilent, CA, USA). To estimate the variation of the gene expression of the RNA of the bacteria post-exposure to carvacrol EO, each sample’s CT was compared with that of the control group (the tested isolates before carvacrol treatment) according to the “ΔΔCt”method described by Yuan et al. [72]. Dissociation curves were compared between the different samples to exclude false-positive results.

## 5. Conclusions

*Staphylococcus* spp. exhibits a high degree of resistance to frequently used antimicrobials. Moreover, an association between the phenotype and genotype for MDR in *Staphylococcus* species was observed. Carvacrol oil inhibited the growth of *Staphylococcus* isolates and unexpectedly, some phenotypic changes were observed with oil treatment. Genotypically, carvacrol induced a reduction in *mec*A and *van*C1 genes’ expression. There is a high correlation between *Staphylococci* isolates from livestock and humans. *Staphylococci* are more likely to spread from animals to humans and vice versa. While a high prevalence of *Staphylococcus* is detected in animals and humans alike, carvacrol and other EOs provide a promising alternative to combat pathogens affecting human health and agriculture production. This is attributed to their potential antimicrobial effect on MDR pathogens; even in sub-lethal doses, this can affect their phenotypic properties and genes expression. 

## Figures and Tables

**Table 1:** Prevalence of *Staphylococcus* species in the different examined samples.

Samples		No. of Samples	Positive Bacterial Isolation	
			No.	%
Cow milk		100	75	75
Sheep abscess		25	13	52
Human samples	Wounds and abscesses	30	22	73.3
	Dermal lesions	33	24	72.7
	Tracheal cavity	28	20	71.4
	Total human samples	91	66	72.5
Overall total		216	154	71.3

%—was calculated according to the corresponding number (No.) of samples.

**Table 2:** Prevalence of different *Staphylococcus* spp. isolates in different examined samples.

Bacterial Isolates	Milk Isolates		Sheep Isolates		Human Isolates		Overall Total No. of Isolates	
	No	%	No	%	No	%	No	%*
*S. aureus*	35	46.7	7	53.8	24	36.4	66	42.9
*S schleiferi*	27	36	2	15.4	10	15.2	39	25.3
*S. intermedius*	8	10.7	2	15.4	9	13.6	19	12.3
*S. xylosus*	2	2.7	1	7.7	15	22.7	18	11.7
*S. haemolyticus*	2	2.7	0	0	5	7.6	7	4.5
*S. epidermidis*	1	1.3	1	7.7	1	1.5	3	1.9
*S. aurecularis*	0	0	0	0	2	3.0	2	1.3
Total No. of isolates	75	100	13	100	66	100	154	100

%—was calculated according to the corresponding total number (No.) of isolates; %*—was calculated according to the overall total number (No.) of isolates (*n* = 154).

**Table 3:** Antibacterial effect of carvacrol oil on MDR *Staphylococcus* isolates.

Bacterial Isolates	No. of Tested Isolates	Carvacrol Oil Concentration											
		0.1%		0.05%		0.04%		0.03%		0.02%		0.01%	
		No	%	No	%	No	%	No	%	No	%	No	%
*S. aureus*	34	34	100	34	100	34	100	25	100	0	0	0	0
*S. schleiferi*	16	16	100	16	100	16	100	12	100	0	0	0	0
*S. intermedius*	8	8	100	8	100	8	100	6	100	0	0	0	0
*S. xylosus*	9	9	100	9	100	9	100	6	100	0	0	0	0
*S. haemolyticus*	4	4	100	4	100	4	100	4	100	0	0	0	0
*S. epidermidis*	1	1	100	1	100	1	100	1	100	0	0	0	0
Total isolates	72	72	100	72	100	72	100	54	75	0	0	0	0
Total non-affected		0	0	0	0	0	0	18	25	72	100	72	100

%—percentage of inhibition that was calculated according to the corresponding number (No.) of tested isolates.

## Data Availability

The data presented in this study are available in Supplementary Materials.

## Supplementary Materials

The following are available online at https://www.mdpi.com/article/10.3390/antibiotics10111328/s1. Table S1: Types of haemolysis and biofilm formation produced by different *Staphylococcus* spp. isolated from different sources; Table S2: Antimicrobial susceptibility of different *Staphylococcus* spp. isolated from cows’ milk samples; Table S3: Antimicrobial susceptibility of different *Staphylococcus* spp. isolated from Sheep abscesses samples; Table S4: Antimicrobial susceptibility of different *Staphylococcus* spp. isolated from human samples; Table S5: Prevalence and distribution of resistance and virulence-associated genes in the examined *Staphylococcus* isolates; Table S6: Primers sequences, target genes, amplicon sizes and cycling conditions for PCR and RT-PCR assays; Table S7: Cycling conditions of the different primers during RT-PCR.
