# Supplementary material for: Carvacrol Essential Oil: A Natural Antibiotic against Zoonotic Multidrug-Resistant Staphylococcus Species Isolated from Diseased Livestock and Humans
Source: Antibiotics (Basel). 2021 Oct 30;10(11):1328. doi: 10.3390/antibiotics10111328 (PMC8614821; doi:10.3390/antibiotics10111328)
Supplement: Supplementary file 1 [file antibiotics-10-01328-s001.zip › antibiotics-1412595-supplementary.pdf]

Supplementary Material

Carvacrol Essential Oil: A Natural Antibiotic against Zoonotic Multidrug-Resistant Staphylococcus Species Isolated from Diseased Livestock and Humans

Ahmed H. Abed [1,*], Esraa F. Hegazy [1], Sherif A. Omar [2], Rehab M. Abd El-Baky [3,4] , Ahmed A. El-Beih [5,*], Ahmed Al-Emam [6,7], Ahmed M.S. Menshawy [8] and Eman Khalifa [9]

[1] Bacteriology, Mycology and Immunology Department, Faculty of Veterinary Medicine, Beni-Suef University, Beni-Suef 62511, Egypt; esraahegazysayed@gmail.com
[2] Microbiology Department, Faculty of Veterinary Medicine, Cairo University, Cairo 12211, Egypt; sherif.marouf@cu.edu.eg
[3] Department of Microbiology and Immunology, Faculty of Pharmacy, Deraya University, Minia 11566, Egypt; rehab.mahmoud@mu.edu.eg
[4] Department of Microbiology and Immunology, Faculty of Pharmacy, Minia University, Minia 61519, Egypt
[5] Chemistry of Natural & Microbial Products Department, National Research Centre, Dokki, Giza 12622, Egypt
[6] Department of Pathology, College of Medicine, King Khalid University, Abha 61421, Saudi Arabia; amalemam@kku.edu.sa
[7] Department of Forensic Medicine and Clinical Toxicology, Faculty of Medicine, Mansoura University, Mansoura 35516, Egypt;
[8] Department of Veterinary Medicine (Infectious Diseases), Faculty of Veterinary Medicine, Beni-Suef University, Beni-Suef 62511, Egypt; elmenshawy81@yahoo.com or ahmed.elmenshawy@vet.bsu.edu.eg
[9] Department of Microbiology, Faculty of Veterinary Medicine, Matrouh University, Matrouh 51511, Egypt; khalifa.eman@alexu.edu.eg
* Correspondence: ahmed.moawad@vet.bsu.edu.eg (A.H.A.); aa.el-beih@nrc.sci.eg (A.A.E.-B.); Tel.: +20-1100878858 (A.H.A.); Tel.: +20-1112914327 (A.A.E.-B.)

Table S1. Types of haemolysis and biofilm formation produced by different *Staphylococcus* spp. isolated from different sources.

Species	Source of Isolates	No. of Tested Isolates	Type of Haemolysis on Sheep Blood Agar						Biofilm Formation					
			α		β		γ		Strong		Intermediate		Total Positive	
			No.	%	No.	%	No.	%	No.	%	No.	%	No.	%
S. aureus	Milk	35	0	0	24	68.6	11	31.4	14	40	5	14.3	19	54.3
	Sheep	7	0	0	5	71.4	2	28.6	4	57.1	0	0	4	57.1
	Human	24	0	0	20	83.3	4	16.7	9	37.5	2	8.3	11	45.8
S. schleiferi	Milk	27	16	59.3	5	18.5	6	22.2	6	22.2	5	18.5	11	40.7
	Sheep	2	2	100	0	0	0	0	0	0	1	50	1	50
	Human	10	5	50	2	20	3	30	3	30	1	10	4	40
S. intermedius	Milk	8	5	62.5	3	37.5	0	0	3	3.5	1	12.5	4	50
	Sheep	2	2	100	0	0	0	0	0	0	0	0	0	0
	Human	9	6	66.7	3	33.3	0	0	3	33.3	1	11.1	4	44.4
S. xylosus	Milk	2	1	50	0	0	1	50	1	50	1	50	2	100
	Sheep	1	0	0	1	100	0	0	0	0	0	0	0	0
	Human	15	3	20	5	33.3	7	46.7	5	33.3	2	13.3	7	46.7
S. haemolyticus	Milk	2	0	0	2	100	0	0	1	50	0	0	1	50
	Human	5	0	0	5	100	0	0	3	60	0	0	3	60
S. epidermidis	Milk	1	0	0	1	100	0	0	0	0	0	0	0	0
	Sheep	1	0	0	1	100	0	0	0	0	0	0	0	0
	Human	1	0	0	1	100	0	0	0	0	1	100	1	100
S. aurecularis	Human	2	0	0	0	0	2	100	0	0	0	0	0	0
Total	Milk	75	22	29.3	35	46.7	18	24	25	33.3	12	16	37	49.3
	Sheep	13	4	30.8	7	53.9	2	15.3	4	30.8	1	7.7	5	38.5
	Human	66	14	21.1	36	54.6	16	24.3	23	34.8	7	10.6	30	45.5
Overall total		154	40	26	78	50.6	36	23.4	52	33.8	20	13	72	46.8

%: was calculated according to the corresponding number (No.) of tested isolates.

Table S2. Antimicrobial susceptibility of different *Staphylococcus* spp. isolated from cows' milk samples.

Antimicrobial Disc	Disc Content (µg)	*S. aureus* (*n*=35)			*S. schleiferi* (*n*=27)			*S. intermedius* (*n*=8)			*S. xylosus* (*n*=2)			*S. haemolyticus* (*n*=2)			*S. epidermedis* (*n*=1)		
		**R**	**I**	**S**	**R**	**I**	**S**	**R**	**I**	**S**	**R**	**I**	**S**	**R**	**I**	**S**	**R**	**I**	**S**
**Ampicillin**	**10**	100	0	0	100	0	0	100	0	0	100	0	0	100	0	0	100	0	0
**Cefoxitin**	**30**	100	0	0	100	0	0	100	0	0	100	0	0	100	0	0	100	0	0
**Vancomycin**	**30**	69	0	31	77	0	23	100	0	0	50	0	50	50	0	50	100	0	0
**Imipenem**	**10**	0	0	100	0	0	100	0	0	100	0	0	100	0	0	100	0	0	100
**Amoxicillin-clavulanic acid**	**5**	100	0	0	100	0	0	100	0	0	100	0	0	100	0	0	100	0	0
**Kanamycin**	**30**	29	57	14	33	52	14	25	75	0	100	0	0	100	0	0	0	0	100
**Clindamycin**	**2**	86	6	8	86	7	7	100	0	0	100	0	0	100	0	0	100	0	0
**azithromycin**	**15**	31	23	46	18	37	44	25	25	50	50	0	50	0	100	0	0	100	0
**Cefuroxime**	**30**	91	6	3	77	15	7	100	0	0	100	0	0	100	0	0	010	0	0
**Chloramphenicol**	**30**	74	6	20	74	7	18	100	0	0	50	0	50	50	0	50	100	0	0
**Sulfamethoxazole-trimethoprim**	**25**	9	11	80	18	4	77	0	25	75	0	0	100	0	0	100	0	100	0
**Ciprofloxacin**	**5**	3	14	82	0	4	96	0	25	75	0	0	100	0	0	100	0	100	0

%: was calculated according to the corresponding number (No.) of tested isolates.

Table S3. Antimicrobial susceptibility of different *Staphylococcus* spp. isolated from Sheep abscesses samples.

Antimicrobial Disc	Disc Content (µg)	*S. aureus* (*n*=7)			*S. schleiferi* (*n*=2)			*S. intermedius* (*n*=2)			*S. xylosus* (*n*=1)			*S. epidermedis* (*n*=1)		
		**R**	**I**	**S**	**R**	**I**	**S**	**R**	**I**	**S**	**R**	**I**	**S**	**R**	**I**	**S**
**Ampicillin**	**10**	100	0	0	100	0	0	100	0	0	100	0	0	100	0	0
**Cefoxitin**	**30**	100	0	0	100	0	0	100	0	0	100	0	0	100	0	0
**Vancomycin**	**30**	71	0	29	50	0	50	0	0	100	100	0	0	100	0	0
**Imipenem**	**10**	0	0	100	0	0	100	0	0	100	0	0	100	0	0	100
**Amoxicillin-clavulanic acid**	**5**	100	0	0	100	0	0	100	0	0	100	0	0	100	0	0
**Kanamycin**	**30**	71	0	29	100	0	0	50	0	50	010	0	0	0	0	100
**Clindamycin**	**2**	100	0	0	100	0	0	100	0	0	100	0	0	100	0	0
**azithromycin**	**15**	29	29	42	0	50	50	0	0	100	0	0	100	0	0	100
**Cefuroxime**	**30**	86	0	14	50	0	50	100	0	0	0	0	100	0	0	100
**Chloramphenicol**	**30**	58	0	42	50	0	50	50	0	50	0	0	100	100	0	0
**Sulfamethoxazole-trimethoprim**	**25**	0	14	86	0	0	100	0	0	100	0	0	100	0	0	100
**Ciprofloxacin**	**5**	0	14	86	0	0	100	0	0	100	0	0	100	0	0	100

%: was calculated according to the corresponding number (No.) of tested isolates.

Table S4. Antimicrobial susceptibility of different *Staphylococcus* spp. isolated from human samples.

Antimicrobial Disc	Disc Content (µg)	*S. aureus* (*n*=24)			*S. schleiferi* (*n*=10)			*S. intermedius* (*n*=9)			*S. xylosus* (*n*=15)			*S. haemolyticus* (*n*=5)			*S. epidermedis* (*n*=1)			*S. aurecularis* (*n*=2)		
		**R**	**I**	**S**	**R**	**I**	**S**	**R**	**I**	**S**	**R**	**I**	**S**	**R**	**I**	**S**	**R**	**I**	**S**	**R**	**I**	**S**
**Ampicillin**	**10**	100	0	0	100	0	0	100	0	0	100	0	0	100	0	0	100	0	0	100	0	0
**Cefoxitin**	**30**	100	0	0	100	0	0	100	0	0	100	0	0	100	0	0	100	0	0	100	0	0
**Vancomycin**	**30**	54	0	46	30	0	70	44	0	56	40	0	60	60	0	40	100	0	0	100	0	0
**Imipenem**	**10**	21	8	71	0	0	100	11	33	56	13	7	80	0	0	100	100	0	0	50	0	50
**Amoxicillin-clavulanic acid**	**5**	100	0	0	100	0	0	100	0	0	100	0	0	100	0	0	100	0	0	100	0	0
**Kanamycin**	**30**	83	17	0	70	20	10	78	11	11	93	0	7	80	20	0	100	0	0	100	0	0
**Clindamycin**	**2**	100	0	0	90	10	0	89	0	11	93	0	7	80	0	20	100	0	0	100	0	0
**Azithromycin**	**15**	50	33	17	60	10	30	78	11	11	67	20	13	80	0	20	100	0	0	100	0	0
**Cefuroxime**	**30**	92	8	0	50	30	20	67	22	11	80	13	7	80	0	20	100	0	0	50	0	05
**Chloramphenicol**	**30**	50	17	33	50	10	40	56	11	33	40	20	40	20	20	60	100	0	0	100	0	0
**Sulfamethoxazole-trimethoprim**	**25**	62	13	25	80	10	10	67	0	33	87	0	13	60	20	20	100	0	0	100	0	0
**Ciprofloxacin**	**5**	54	13	33	70	20	10	67	11	22	80	0	20	40	0	60	100	0	0	100	0	0

%: was calculated according to the corresponding number (No.) of tested isolates.

Table S5. Prevalence and distribution of resistance and virulence-associated genes in the examined *Staphylococcus* isolates.

Species	Source of Isolates	No. of Tested Isolate	Resistance Genes								Virulence Genes											
			*mec*A		*van*A		*van*C1		*erm*C		*hla*		*ica*A		*ica*D		*spa*		*sea*		*sed*	
			**No.**	**%**	**No.**	**%**	**No**	**%**	**No.**	**%**	**No.**	**%**	**No.**	**%**	**No.**	**%**	**No.**	**%**	**No.**	**%**	**No.**	**%**
S. aureus	Milk	19	19	100	11	57.9	17	89.5	3	15.9	11	57.9	15	78.9	17	89.5	14	73.7	0	0	0	0
	Sheep	4	4	100	4	100	4	100	1	25	3	75	3	75	4	100	3	75	0	0	0	0
	Human	11	11	100	8	72.7	10	90.1	3	27.3	7	63.6	10	90.1	11	100	8	72.7	0	0	0	0
S. schleiferi	Milk	11	10	90.9	4	36.4	8	72.7	0	0	3	27.3	8	72.7	7	63.6						
	Sheep	1	0	0	0	0	1	100	0	0	0	0	1	100	1	100						
	Human	4	3	75	0	0	3	75	0	0	2	50	3	75	3	75						
S. intermedius	Milk	4	3	75	2	66.7	2	50	0	0	2	50	2	50	1	25						
	Human	4	3	75	0	0	3	75	0	0	2	50	3	75	2	50						
S. xylosus	Milk	2	2	100	1	50	0	0	1	50	0	0	2	100	2	100						
	Human	7	7	100	0	0	6	85.7	0	0	3	42.9	3	42.9	3	42.9	Not investigated					
S. haemolyticus	Milk	1	1	100	0	0	1	100	0	0	0	0	1	100	1	100						
	Human	3	3	100	0	0	3	100	0	0	2	66.7	3	100	3	100						
S. epidermidis	Human	1	1	100	0	0	1	100	1	100	1	100	0	0	1	100						
Total	Milk	37	35	94.6	18	48.6	29	78.4	4	10.8	16	43.2	28	75.7	28	75.7						
	Sheep	5	4	80	4	80	5	100	1	20	3	60	4	80	5	100						
	Human	30	28	93.3	8	26.7	26	86.7	4	13.3	17	56.7	22	73.3	23	76.7						
Overall total		72	67	93.1	30	41.7	60	83.3	9	12.5	36	50	54	75	56	77.8						

%: was calculated according to the corresponding number (No.) of tested isolates.

Table S6. Primers sequences, target genes, amplicon sizes and cycling conditions for PCR and RT-PCR assays.

Primers	Primers Sequences	Amplified Product	Primary Denaturation	Amplification (35 Cycles)			Final Extension	References
				2ry Denaturation	Annealing	Extension		
16S rRNA	*CCTATAAGACTGGGATAACTTCGGG*<br>*CTTTGAGTTTCAACCTTGCGGTCG*	791 bp	94°C<br>5 min.	94°C<br>30 sec.	55°C<br>40 sec.	72°C<br>45 sec.	72°C<br>10 min.	[61]
*mec*A	*GTAGAAATGACTGAACGTCCGATAA*<br>*CCAATTCCACATTGTTTCGGTCTAA*	310 bp	94°C<br>5 min.	94°C<br>30 sec.	50°C<br>30 sec.	72°C<br>30 sec.	72°C<br>7 min.	[62]
*van*A	*CATGACGTATCGGTAAAATC*<br>*ACCGGGCAGRGTATTGAC*	885 bp	94°C<br>5 min.	94°C<br>30 sec.	50°C<br>40 sec.	72°C<br>45 sec.	72°C<br>10 min.	[63]
*van*C1	*GGTATCAAGGAAACCTC*<br>*CTTCCGCCATCATAGCT*	822 bp	94°C<br>5 min.	94°C<br>30 sec.	54°C<br>40 sec.	72°C<br>45 sec.	72°C<br>10 min.	[64]
*erm*C	*ATCTTTGAAATCGGCTCAGG*<br>*CAAACCCGTATTCCACGATT*	299 bp	94°C<br>5 min.	94°C<br>30 sec.	51°C<br>30 sec.	72°C<br>30 sec.	72°C<br>7 min.	[65]
hla	*GAAGTCTGGTGAAAACCCTGA*<br>*TGAATCCTGTCGCTAATGCC*	704 bp	94°C<br>5 min.	94°C<br>30 sec.	53°C<br>40 sec.	72°C<br>45 sec.	72°C<br>10 min.	[66]
*ica*A	*CCTAACTAACGAAAGGTAG*<br>*AAGATATAGCGATAAGTGC*	1315 bp	94°C<br>5 min.	94°C<br>30 sec.	49°C<br>1 min.	72°C<br>1 min.	72°C<br>12 min.	[67]
*ica*D	*AAACGTAAGAGAGGTGG*<br>*GGCAATATGATCAAGATA*	381 bp	94°C<br>5 min.	94°C<br>30 sec.	49°C<br>30 sec.	72°C<br>30 sec.	72°C<br>7 min.	
spa	*TCAACAAAGAACAACAAAATGC*<br>*GCTTTCGGTGCTTGAGATTC*	226 bp	94°C<br>5 min.	94°C<br>30 sec.	55°C<br>30 sec.	72°C<br>30 sec.	72°C<br>7 min.	[68]
sea	*TTGGAAACGGTTAAAACGAA*<br>*GAACCTTCCCATCAAAAACA*	120 bp	94°C<br>5 min.	94°C<br>30 sec.	50°C<br>30 sec.	72°C<br>30 sec.	72°C<br>7 min.	[69]
sed	*CCAATAATAGGAGAAAATAAAAG*<br>*ATTGGTATTTTTTTTCGTTC*	278 bp	94°C<br>5 min.	94°C<br>30 sec.	57°C<br>40 sec.	72°C<br>45 sec.	72°C<br>10 min.	[70]

Table S7. Cycling conditions of the different primers during RT-PCR.

Target Gene	Reverse Transcription	Primary Denaturation	Amplification (40 Cycles)			Dissociation Curve (1 Cycle)		
			Secondary Denaturation	Annealing (Optics on)	Extension	Secondary Denaturation	Annealing	Final Denaturation
16S *rRNA*	50˚C/ 30 min.	94˚C/ 15 min.	94˚C/ 15 sec.	55˚C/ 30 sec.	72˚C/ 40 sec.	94˚C/ 1 min.	55˚C/ 1 min.	94˚C/ 1 min.
*mec*A				50˚C/ 30 sec.			50˚C/ 1 min.	
*van*C1				54˚C/ 30 sec.			54˚C/ 1 min.	
